# Dehydration accelerates reductions in cerebral blood flow during prolonged exercise in the heat without compromising brain metabolism

**DOI:** 10.1152/ajpheart.00525.2015

**Published:** 2015-09-14

**Authors:** Steven J. Trangmar, Scott T. Chiesa, Iñaki Llodio, Benjamin Garcia, Kameljit K. Kalsi, Niels H. Secher, José González-Alonso

**Affiliations:** ^1^Centre for Sports Medicine and Human Performance, Brunel University London, Uxbridge, United Kingdom; and; ^2^Department of Anaesthesia, The Copenhagen Muscle Research Centre, Rigshospitalet, University of Copenhagen, Denmark

**Keywords:** cerebral blood flow, dehydration, extracranial blood flow, prolonged exercise

## Abstract

Dehydration hastens the decline in cerebral blood flow (CBF) during incremental exercise, whereas the cerebral metabolic rate for O_2_ (CMR_O_2__) is preserved. It remains unknown whether CMR_O_2__ is also maintained during prolonged exercise in the heat and whether an eventual decline in CBF is coupled to fatigue. Two studies were undertaken. In *study 1*, 10 male cyclists cycled in the heat for ∼2 h with (control) and without fluid replacement (dehydration) while internal and external carotid artery blood flow and core and blood temperature were obtained. Arterial and internal jugular venous blood samples were assessed with dehydration to evaluate CMR_O_2__. In *study 2*, in 8 male subjects, middle cerebral artery blood velocity was measured during prolonged exercise to exhaustion in both dehydrated and euhydrated states. After a rise at the onset of exercise, internal carotid artery flow declined to baseline with progressive dehydration (*P* < 0.05). However, cerebral metabolism remained stable through enhanced O_2_ and glucose extraction (*P* < 0.05). External carotid artery flow increased for 1 h but declined before exhaustion. Fluid ingestion maintained cerebral and extracranial perfusion throughout nonfatiguing exercise. During exhaustive exercise, however, euhydration delayed but did not prevent the decline in cerebral perfusion. In conclusion, during prolonged exercise in the heat, dehydration accelerates the decline in CBF without affecting CMR_O_2__ and also restricts extracranial perfusion. Thus, fatigue is related to a reduction in CBF and extracranial perfusion rather than CMR_O_2__.

## Abstract (precis)

*Reductions in cerebral blood flow and extracranial perfusion, induced by dehydration during prolonged exercise in the heat, may be coupled to fatigue. However, cerebral metabolism remains stable through enhanced O_2_ and glucose extraction. Thus, fatigue developed during prolonged exercise with dehydration is related to reductions in cerebral blood flow rather than to the cerebral metabolic rate for O_2_*.

## NEW & NOTEWORTHY

*Reductions in cerebral blood flow and extracranial perfusion, induced by dehydration during prolonged exercise in the heat, may be coupled to fatigue. However, cerebral metabolism remains stable through enhanced O_2_ and glucose extraction. Thus, fatigue developed during prolonged exercise with dehydration is related to reductions in cerebral blood flow rather than to the cerebral metabolic rate for O_2_*.

dehydration and hyperthermia accrued during prolonged exercise in the heat pose a challenge to cardiovascular control, evidenced by reductions in stroke volume, cardiac output, skeletal muscle and skin blood flow, and, to a lesser extent, mean arterial pressure (MAP) (12, 16, 29). The cardiovascular strain imposed by combined dehydration and hyperthermia might also encompass cerebral blood flow (CBF) as an orthostatic challenge (35), pharmacological interventions that depress cardiac output (19), passive heat stress (2, 31), and combined heat stress and exercise (34) compromise cerebral perfusion. However, the role of hydration on the effect of prolonged exercise-induced dehydration on CBF and cerebral metabolism [cerebal metabolic rate of O_2_ (CMR_O_2__)] is unknown.

Reductions in O_2_ and substrate supply can compromise organ and tissue metabolism (13) and the circulatory strain induced by dehydration during prolonged exercise in a hot environment may compromise cerebral substrate delivery to an extent that impairs CMR_O_2__ and central nervous system function, ultimately curtailing performance. CMR_O_2__ is maintained during incremental exercise (44, 15), but it remains unknown whether CBF and CMR_O_2__ are preserved during prolonged exercise in the heat or whether eventual deviations relate to fatigue.

Thermoregulatory processes increase and modify the distribution of skin and deep tissue blood flow (6, 7) to regulate body temperature during exercise, particularly during heat stress (14). Across the head circulation, increasing skin and body temperatures lead to progressive elevations in external carotid artery (ECA) blood flow, as evidenced during incremental exercise in normothermia (40) and with passive heat stress (2, 36). The ECA serves the cutaneous circulation of the neck and face, which is important for heat liberation. In contrast, CBF progressively declines at high exercise intensities and with high body temperatures (36, 41). Altered perfusion pressure, blood gas tensions [particularly arterial Pco_2_ (Pa_CO_2__)] (48) and sympathetic activity (28) have been implicated in the control of CBF, whereas body temperature is known to influence ECA blood flow (36, 40, 41). However, no study has, as yet, characterized the regional distribution of blood flow across the head during prolonged exercise with and without progressive reductions in total body water.

The aim of the present study, therefore, was to assess the effect of dehydration on cerebral and extracranial hemodynamics and CMR_O_2__ during prolonged exhaustive exercise in the heat. A second aim was to gain insights into the potential mechanisms underlying CBF responses to dehydration and prolonged exercise during nonexhaustive and exhaustive exercise. We hypothesized that dehydration accrued during prolonged exercise in the heat would reduce CBF but that CMR_O_2__ would be maintained. A second hypothesis was that ECA blood flow would increase in relation to increasing core temperature. Finally, we hypothesized that the cerebral circulatory strain occurring during strenuous exercise in the heat is an epiphenomenon of fatigue rather than the direct consequence of dehydration.

## METHODS

Fully informed written consent was obtained from the participants before the studies. All procedures were approved by the Brunel University London Research Ethics Committee (RE07-11 and RE20-09) and conformed with guidelines of the World Medical Association (Declaration of Helsinki).

Ten healthy endurance-trained male subjects [means ± SD; age: 29 ± 5 yr, height: 183 ± 5 cm, body mass: 78 ± 9 kg, and maximal O_2_ consumption (V̇o_2 max_): 59 ± 6 ml·kg^−1^·min^−1^] participated in *study 1*. In *study 2*, the age, height, body mass, and V̇o_2 max_ of the eight endurance-trained male subjects were 33 ± 4 yr, 173 ± 4 cm, 75 ± 11 kg, and 56 ± 7 ml·kg^−1^·min^−1^, respectively. All participants arrived at the laboratory with normal hydration status and were required to have abstained from alcohol intake for 24 h and caffeine consumption for 12 h.

### 

#### Experimental protocols.

The experimental design of *study 1* has been previously described (44). Participants visited the laboratory for 3 preliminary trials followed by 2 experimental trials, each separated by at least 1 wk. During the preliminary trials, participants were familiarized with the methodology before completing incremental exercise on a semirecumbent cycle ergometer (Lode Angio, Groningen, The Netherlands), with a backrest inclination of 45°, to establish the maximal work rate, maximal heart rate (HR), and V̇o_2 max_. Participants were then familiarized to the heat and experimental protocol by cycling in an environmental chamber set at 35°C (relative humidity: 50%) in the semirecumbent position for 2 h at 55% of the maximal work rate with HR and intestinal temperature recorded. No fluid consumption was permitted during exercise. To determine hydration status, body mass was recorded before and immediately after exercise in all trials.

On the 2 experimental trials (*visits 4* and *5*), participants performed prolonged (∼2 h) continuous cycling exercise after an initial incremental exercise test and 1 h of rest. In the first experimental trial, participants were not permitted to consume fluid during the prolonged exercise, whereas on the second experimental trial (i.e., control trial), participants completed the same exercise protocol but hydration was maintained through fluid ingestions according to the participants' body mass loss during the previous visit. Fluid was provided in aliquots of ∼200 ml every 10 min during exercise. Both experimental trials were performed in the heat (same conditions as in the familiarization sessions), and pedal cadence was maintained at 70–90 rpm.

In the dehydration trial, CBF and blood samples from the brachial artery and left internal jugular vein were obtained simultaneously at rest and every 30 min during exercise. Core, skin, and jugular venous temperatures and arterial and jugular venous pressures were recorded continuously. The same measures were collected in the control trial except for the arterial and internal jugular venous blood sampling and intra-arterial/venous blood pressures.

A similar experimental design including five laboratory visits separated by a week was used in *study 2*. During the experimental trials, however, participants cycled in the heat (35°C, 50% relative humidity, and fan cooling) at 60% V̇o_2 max_ until volitional exhaustion in the dehydration and euhydration conditions while HR, core and skin temperatures, and middle cerebral artery (MCA) blood velocity (*V*_mean_) were monitored.

#### CBF.

Vessel blood flow was obtained sequentially at rest and every 30 min from the right internal carotid artery (ICA), ECA, and common carotid artery (CCA) using an ultrasound system (Vivid 7 Dimension, GE Healthcare) equipped with a 10-MHz linear array transducer. ICA, ECA, and CCA measurements were typically obtained ∼1.0–1.5 cm above and ∼1.5 cm below the carotid bifurcation, respectively, and the coefficients of variation for measurements of ICA, ECA, and CCA vessel diameter and volume flow were considered within an acceptable range both at rest (2.8 ± 0.9%, 2.1 ± 1.1%, and 4.3 ± 1.0%) and during exercise (5.3 ± 1.6%, 5.1 ± 1.4%, and 5.0 ± 1.6%). For the calculation of blood flow, two-dimensional brightness mode images for vessel diameter were obtained, and the mean diameter was calculated as systolic diameter × 1/3 + diastolic diameter × 2/3. Time-averaged mean flow *V*_mean_ (in cm/s) was measured continuously in pulse wave mode over 60 s. Throughout blood flow measurements, care was made to ensure a consistent insonation angle below 60°, and the sample volume was maintained at the center of the vessel lumen and adjusted to cover its width. Mean flow velocity profiles were traced automatically and analyzed offline for the determination of time-averaged mean flow velocity (EchoPAC BT12, version 112, GE Healthcare). Blood flow (in ml/min) was then calculated using mean flow velocity × cross-sectional area [CSA; where CSA = π × (mean diameter/2)^2^ and blood flow = time-averaged mean flow velocity × CSA × 60]. MCA *V*_mean_ was measured using a 2-MHz pulsed transcranial Doppler ultrasound system (DWL, Sipplingen, Germany). The right MCA was insonated through the temporal ultrasound window, distal to the MCA-anterior cerebral artery bifurcation, at a depth of 45–60 mm. Signal quality was optimized according to Aaslid et al. (1).

#### Catheter placement and blood sampling.

Catheters for blood sampling, arterial pressure, internal jugular venous pressure, and blood temperature were inserted into the brachial artery of the nondominant arm and, after local anesthesia (2% lidocaine), in the left internal jugular vein (Double Lumen Catheter, 16 gauge, 2.3 mm, Multi-Med M2716HE, Edwards Lifesciences) using the Seldinger technique, and the catheter was advanced to the jugular bulb. For measurements of jugular venous blood temperature, a thermistor (T204-D, PhysiTemp, Clifton, NJ) was inserted through the catheter and connected to a thermocouple meter (TC-2000, Sable Systems). The internal jugular catheter was inserted under ultrasound guidance, and catheters were flushed with 0.9% saline to maintain patency. An ∼1-h period of rest was observed between catheterization and the commencement of resting measurements.

#### Blood variables.

Arterial and jugular venous blood samples were drawn into preheparinized syringes and analyzed immediately for blood gas variables (ABL 800 FLEX, Radiometer, Copenhagen, Denmark), corrected for blood temperature in the internal jugular vein. The analyzer was calibrated at regular intervals in accordance with manufacturer guidelines. Additional arterial and jugular venous blood were collected in 2-ml syringes, transferred to EDTA tubes, centrifuged, and separated. Plasma epinephrine and norepinephrine (NE) were subsequently determined using an enzyme-linked immunoassay kit (DEE6500 2-CAT, Demeditec Diagnostics).

#### HR, blood pressure, and temperature.

HR was obtained by telemetry (Polar Electro, Kempele, Finland). Arterial and internal jugular venous pressure waveforms were recorded using transducers (Pressure Monitoring Kit, TruWave, Edwards Lifesciences) zeroed at the level of the right atrium in the midaxillary line (arterial) and at the level of the tip of the catheter (jugular venous). During the control trial, reconstructed brachial artery pressure waveforms were obtained noninvasively (Finometer Pro, Finapress Medical Systems). Arterial pressure waveforms were sampled at 1,000 Hz, amplified (BP Amp, ADInstruments, Oxfordshire, UK), and connected to a data-acquisition unit (Powerlab 16/30, ADInstruments) for offline analysis. Intestinal temperature was measured using an ingestible telemetry pill (HQ, Palmetto, FL), and mean skin temperature was obtained using a wired thermocouple system (TC-2000, Sable Systems) from standard weightings of chest, abdomen, thigh, and calf temperatures (38).

#### Calculations.

Cerebral vascular conductance indexes were calculated by dividing blood flow in the ICA, ECA, CCA, and MCA *V*_mean_ by MAP. Arterial O_2_ content was used to quantify O_2_ delivery through the ICA and MCA, respectively. CMR_O_2__ and cerebral glucose and lactate uptake were calculated as ICA flow × 2, multiplied by the corresponding arterial to venous difference. The molar ratio of O_2_ to glucose [O_2_-to-glucose index (OGI)] and O_2_-to-carbohydrate index (OCI; O_2_/glucose + ½lactate index) were also calculated.

#### Statistical analysis.

All analyses were made using IBM SPSS Statistics (version 20, IBM, Armonk, NY). Variables were assessed using two-way repeated-measures ANOVA in which condition (dehydration and control) and exercise phase (rest, 30, 60, 90, and 120 min) were the main factors, with the Dunn-Sidak correction used for multiple comparisons. Multiple regression for within-subject repeated measures was used for analysis of the relationship between blood flow and blood gas variables and temperature (3). Data are presented as means ± SE, and the α-level for significance was set at *P* < 0.05.

## RESULTS

### 

#### Hydration and temperature.

Exercise without supplementation of fluid resulted in a 3% body mass reduction (78.2 ± 2.7 to 75.8 ± 2.7 kg) and a 10-min reduction in exercise duration (110 ± 2 vs. 120 min in the control trial, both *P* < 0.001). In the control trial, body mass was maintained at the preexercise level (79 ± 3 kg) through the consumption of fluid (∼1.2 l/h). The decline in body mass with dehydration was accompanied by increases in arterial and venous hemoglobin content (*P* < 0.05), indicative of plasma volume reductions. Intestinal temperature increased progressively in both trials but was higher at the end of exercise in the dehydration trial compared with the control trial (38.7 ± 0.1 vs. 38.2 ± 0.2°C, *P* < 0.05; Table 1). Internal jugular venous blood temperature mirrored the rise in intestinal temperature in the dehydration trial (see Fig. 4*B*). Mean skin temperature was maintained throughout exercise in both dehydration and control trials (∼32.8 and ∼32.6°C; Table 1). HR was similar at rest but during the second h of exercise was maintained at ∼14 beats/min higher in the dehydration trial compared with the control trial (*P* < 0.05).

**Table 1.** Temperature and heart rate responses to dehydration and euhydration (control) during prolonged exercise

	Cycling Time, min				
	Rest	30	60	90	End exercise
Intestinal temperature, °C					
Dehydration	37.4 ± 0.1	38.0 ± 0.1*	38.4 ± 0.1*‡	38.6 ± 0.1*‡	38.7 ± 0.1*‡
Control	37.3 ± 0.1	37.9 ± 0.1*	38.1 ± 0.1*	38.2 ± 0.1*	38.2 ± 0.2*
Mean skin temperature, °C					
Dehydration	34.0 ± 0.3	33.1 ± 0.4	32.7 ± 0.4	32.8 ± 0.4	32.6 ± 0.3
Control	33.5 ± 0.3	32.6 ± 0.3	32.6 ± 0.3	32.8 ± 0.3	32.3 ± 0.3
Heart rate, beats/min					
Dehydration	80 ± 3	148 ± 2*‡	157 ± 2*†‡	163 ± 2*†‡	166 ± 3*†‡
Control	77 ± 2	142 ± 3*	145 ± 3*	149 ± 3*†	149 ± 3*†

Values are means ± SE for 10 participants. Data presented are from the dehydration trial only.

* *P*< 0.05 vs. rest;

† *P*< 0.05 vs. 30 min;

‡ *P*< 0.05 vs. control.

#### Brain and extracranial hemodynamics and metabolism.

**Fig. 1.**
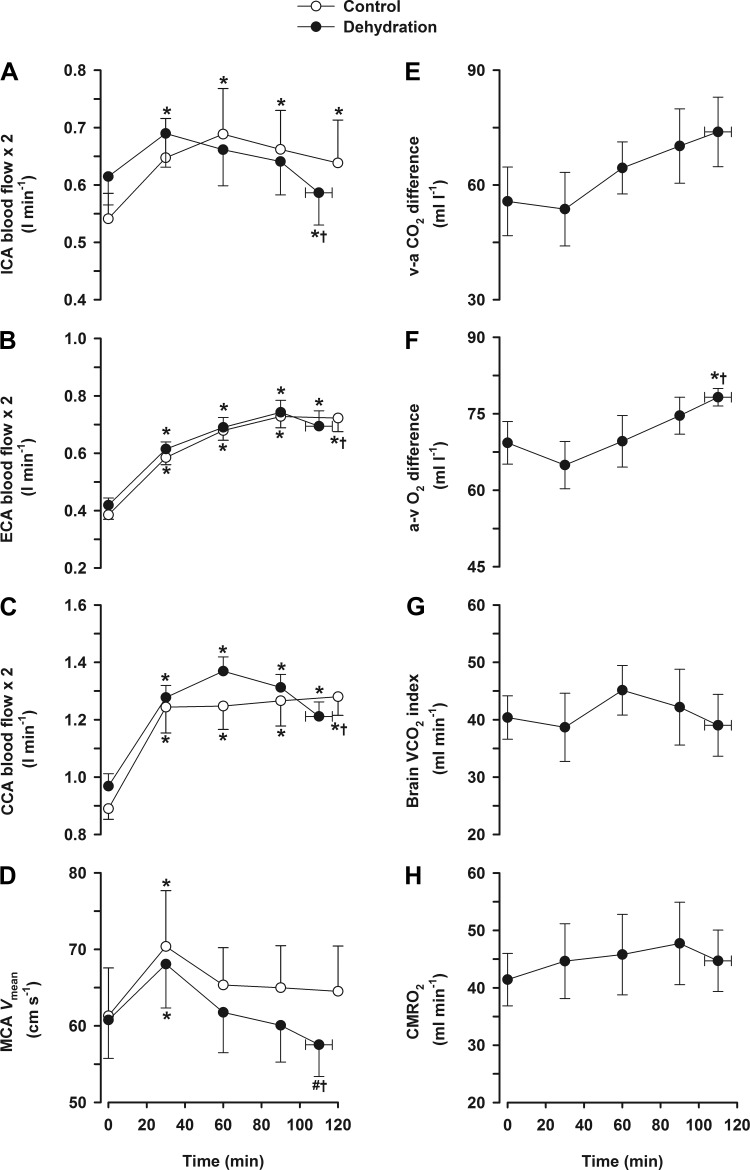
Cerebral and extracerebral hemodynamics and O_2_ parameters during prolonged exercise. Values are means ± SE for 10 participants. Cerebral hemodynamics were obtained in both the dehydration and control exercise trials (*A–D*) but not for O_2_/CO_2_ parameters (*E–H*). *A*: internal carotid artery (ICA) blood flow. *B*: external carotid artery (ECA) blood flow. *C*: common carotid artery (CCA) blood flow. *D*: middle cerebral artery (MCA) mean blood velocity (*V*_mean_). *E*: venous to arterial (v-a) CO_2_ difference. *F*: arterial to venous (a-v) O_2_ difference. *G*: brain CO_2_ output (V̇co_2_) index. *H*: cerebral metabolic rate of O_2_ (CMR_O_2__). *P < 0.05 vs. rest; †*P* < 0.05 vs. the 30-min value.

In the dehydration trial, ICA blood flow and MCA *V*_mean_ increased by ∼12% at 30 min (*P* < 0.05) before declining progressively to baseline values at the end of exercise (*P* < 0.05; Fig. 1, *A* and *D*). The decline in ICA blood flow was associated with a reduction in blood flow velocity (*P* < 0.05) but not in vessel diameter. On the other hand, in the control trial, ICA blood flow and MCA *V*_mean_ increased and remained stable throughout exercise. During the dehydration trial, ECA (and CCA) blood flow almost doubled from rest to 90 min (0.42 ± 0.03 to 0.74 ± 0.04 l/min) but then declined at the end of exercise (*P* < 0.05; Fig. 1, *B* and *C*). In contrast, during the control trial, ECA flow increased similarly up to 90 min of exercise and then plateaued.

**Fig. 2.**
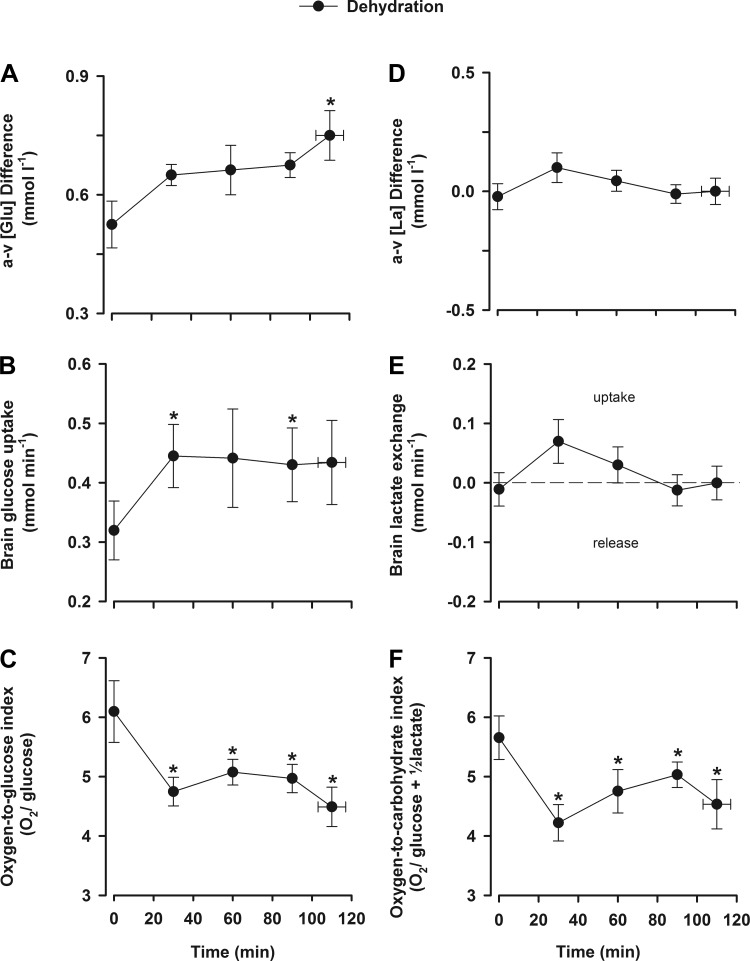
Cerebral lactate and glucose exchange during prolonged exercise. Values are means ± SE for 10 participants. Data presented are from the dehydration trial. *A*: a-v glucose concentration ([Glu]) difference. *B*: brain glucose uptake. *C*: O_2_-to-glucose index. *D*: a-v lactate concentration ([La]) difference. *E*: brain lactate exchange. *F*: O_2_-to-carbohydrate index. *P < 0.05 vs. rest.

The decline in ICA blood flow at the end of dehydration exercise was accompanied by an increased arterial to venous O_2_ difference (*P* < 0.05) but no changes in the arterial to venous CO_2_ difference or brain CO_2_ output index. Thus, CMR_O_2__ was stable throughout exercise. Both arterial and jugular venous plasma glucose gradually declined throughout prolonged exercise (5.4 ± 0.2 to 5.1 ± 0.2 and 5.4 ± 0.2 to 4.4 ± 0.2 mmol/l, respectively, *P* < 0.05). However, the brain arterial to venous glucose difference was stable during the early stages of exercise before increasing before the end of exercise (peak value of 0.7 mmol/l, *P* < 0.05; Fig. 2*A*), whereas brain glucose uptake increased initially (0.33 to 0.43 mmol/min, *P* < 0.05; Fig. 2*B*) and then remained stable.

At rest, the brain released a small amount of lactate (0.2 ± 0.05 mmol/l), and during prolonged exercise with dehydration, arterial and jugular venous lactate gradually declined (3.4 ± to 2.4 ± 0.3 and 3.6 ± 0.5 to 2.4 ± 0.3 mmol/l, *P* < 0.05). The brain arterial to venous lactate difference was maintained throughout exercise, as was lactate exchange (Fig. 2, *D* and *E*). The molar ratio of O_2_ to glucose declined at the onset of exercise (6.1 ± 0.5 vs. 4.5 ± 0.3, *P* < 0.05; Fig. 2*C*) and thereafter remained stable, with a similar response when lactate metabolism was accounted for (Fig. 2*F*).

#### Brain and extracranial vascular conductance.

**Fig. 3.**
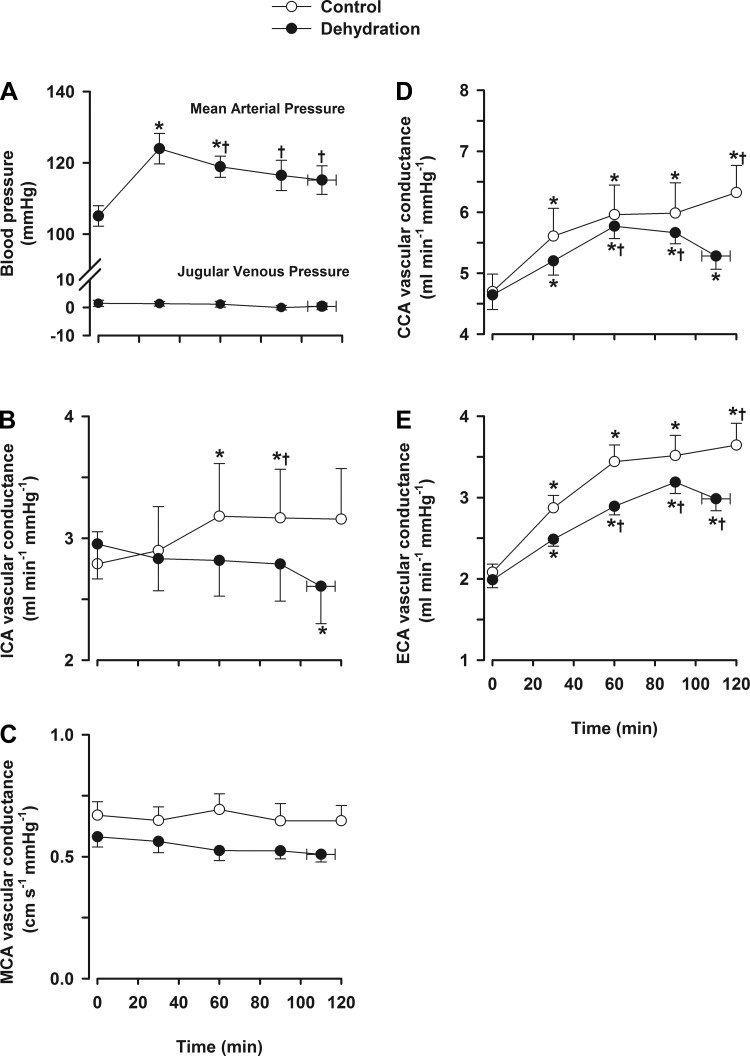
Blood pressure and cerebral and extracerebral vascular conductance during prolonged exercise. Values are means ± SE for 10 participants. *A*: blood pressure. *B*: ICA vascular conductance. *C*: MCA vascular conductance. *D*: CCA vascular conductance. *E*: ECA vascular conductance. *P < 0.05 vs. rest; †*P* < 0.05 vs. the 30-min value.

In the dehydration trial, MAP increased ∼18% from rest to 30 min (105 ± 3 to 124 ± 4 mmHg, *P* < 0.05) before declining progressively until exercise termination (*P* < 0.05). In the control trial, MAP increased by 11% from rest to 30 min and then remained stable. During prolonged exercise, ICA and MCA *V*_mean_ vascular conductance were lower in the dehydration trial compared with the control trial (*P* < 0.05). At the end of exercise in the dehydration trial, both ICA and MCA *V*_mean_ vascular conductance indexes were reduced (*P* < 0.05; Fig. 3), whereas in the control trial, they remained stable. CCA and ECA vascular conductance were similar between trials during early exercise but were reduced at the end of exercise in the dehydration trial compared with the control trial (*P* < 0.05).

#### Blood flow and Pa_CO_2__, plasma catecholamines, and temperature.

**Fig. 4.**
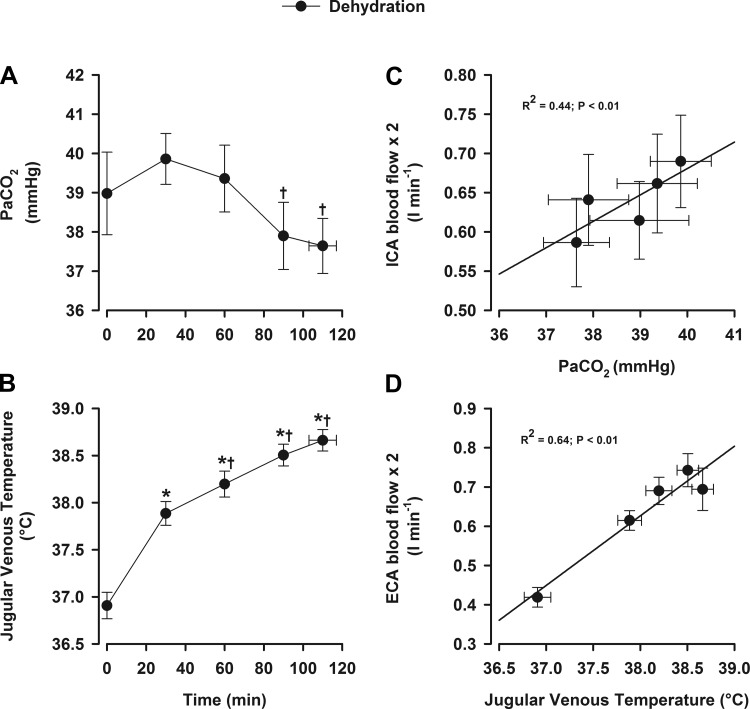
Blood temperature, arterial Pco_2_ (Pa_CO_2__), and relationships with blood flow during prolonged exercise. Values are means ± SE for 10 participants. Data presented are from the dehydration trial. Relationships were obtained using multiple regression for within-subject repeated measures. *A*: Pa_CO_2__. *B*: jugular venous temperature. *C*: ICA blood flow. *D*: ECA blood flow. *P < 0.05 vs. rest; †*P* < 0.05 vs. the 30-min value.

In the dehydration trial, Pa_CO_2__ declined to below baseline at the end of exercise (∼6% reduction from the peak value, *P* < 0.05; Fig. 4*A*), whereas venous Pco_2_ remained unchanged throughout exercise. During exercise, the decline in both ICA blood flow (*R*^*2*^ = 0.44; Fig. 4*C*) and MCA *V*_mean_ (*R*^*2*^ = 0.5; data not shown) were correlated with reduced Pa_CO_2__ (both *P* < 0.01) and, to a weaker extent, also with arterial NE concentration (*R*^*2*^ = 0.15, *P* < 0.05) but not to with jugular venous NE concentration (*R*^*2*^ = 0.02, *P* = 0.58).

In the dehydration trial, arterial epinephrine concentraton increased from rest to 30 min (0.7 ± 0.2 to 1.1 ± 0.2 nmol/l, *P* < 0.05) but remained stable throughout exercise (range: 1.1–2.7 nmol/l), whereas jugular venous epinephrine concentration did not increase until after 90 min of exercise (110 ± 2 min = 2.3 ± 0.7 nmol/l, *P* < 0.05 vs. rest). Arterial NE concentration increased to a peak of 33 ± 8.2 nmol/l (*P* < 0.05 vs. rest), whereas jugular venous NE concentration increased to 90 min (3.6 ± 1.2 to 31.0 ± 5.2 nmol/l, *P* < 0.01) before declining before exhaustion (14.1 ± 1.2 nmol/l), indicating the cerebral uptake of catecholamines at exhaustion.

The alterations in ECA blood flow and vascular conductance were associated with increases in internal jugular venous blood temperature (*R*^*2*^ = 0.48, *P* < 0.01; Fig. 4, *B* and *D*) and jugular venous NE concentration (*R*^*2*^ = 0.48, *P* < 0.01). However, a consistent relationship with blood temperature was not observed beyond 90 min, where the 7% decline in ECA blood flow (and conductance) was matched with an increased blood temperature (Fig. 4*B*).

#### Hydration, core temperature, and the cerebral circulation during exhaustive exercise.

Hydration status was the same before exercise, as indicated by the same body mass in both trials (75.2 ± 4.2 and 75.3 ± 4.2 kg). When participants did not ingest fluids during exercise, they became progressively dehydrated to a 3.2 ± 0.3% reduction in body mass, whereas they maintained body mass in the euhydration trial (+0.3 ± 0.2% body mass). In addition, they became exhausted sooner in the dehydrated state than when euhydrated (123 ± 7 vs. 145 ± 9 min, *P* < 0.05), with higher core temperature and HR at exhaustion (38.7 ± 0.2 vs. 38.3 ± 0.1°C and 156 ± 4 vs. 148 ± 5 beats/min, both *P* < 0.05). Core and skin temperatures and HR, however, were similar between trials during the first 40–50 min of exercise. MCA *V*_mean_ increased during the first 30 min of the exercise and thereafter declined in both trials (*P* < 0.05 vs. the 30-min value).

## DISCUSSION

The main finding of the present study is that dehydration impaired prolonged exercise capacity in the heat in association with an accelerated reduction in CBF. However, despite the reduced CBF, cerebral metabolism was preserved by elevated O_2_ and glucose extraction from blood. A second novel finding was that in both experimental trials, the rise in ECA blood flow was attenuated before volitional exhaustion. The reduction in CBF was related to reduced Pa_CO_2__, whereas increased ECA vascular conductance was associated with increasing internal temperature. Although maintenance of euhydration by fluid ingestion preserved cerebral and extracranial blood flow throughout nonfatiguing exercise, it only delayed the decline in CBF during exhaustive exercise. These findings suggest the CBF decline with dehydration is coupled to factors associated with the fatigue processes rather than dehydration per se.

### 

#### Cerebral and extracranial hemodynamics.

**Fig. 5.**
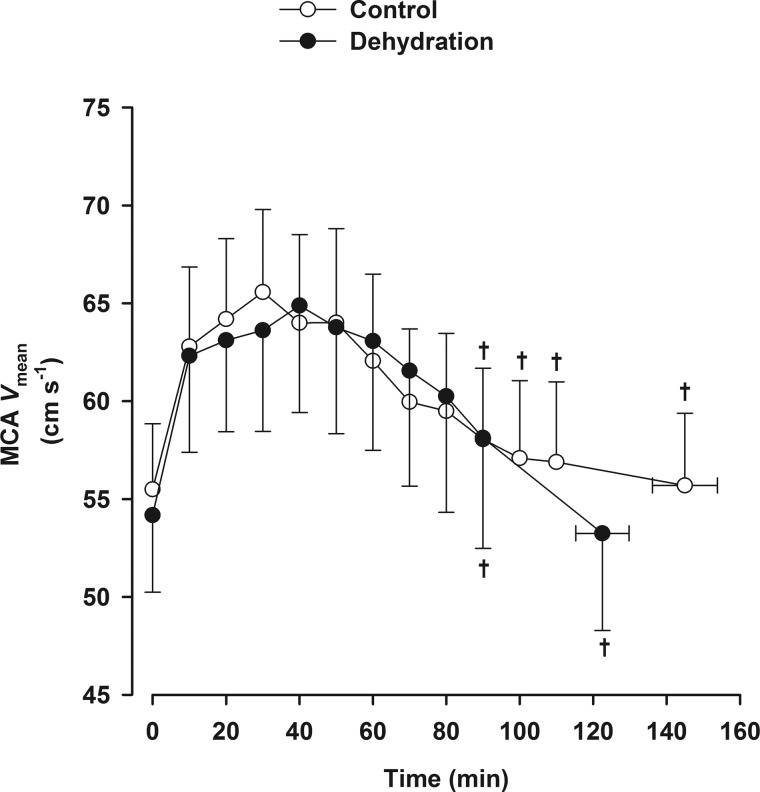
MCA *V*_mean_ with and without dehydration during prolonged exercise to exhaustion. Values are means ± SE for 8 participants. MCA *V*_mean_ declined after 30 min of exercise in both trials. †*P* < 0.05 vs. the 30-min value.

A first aim of the study was to characterize hemodynamic responses of the cerebral and extracranial circulations to dehydration during prolonged exercise in the heat. We found that after the well-established increase upon the onset of exercise, CBF and MCA *V*_mean_ declined gradually to resting values at volitional exhaustion concomitant with the development of dehydration. Conversely, when hydration was maintained during similar duration exercise, CBF did not decline. Similarly, CBF remains stable during prolonged exercise in a thermoneutral environment when the degree of dehydration is negligible (33, 34). However, when exercise in a warm environment causes hyperthermia and cardiovascular strain, CBF is suppressed (37) before declining to resting values (34). These studies, however, did not establish whether hyperthermia, dehydration, or other factors underpinning volitional exhaustion are responsible for the fall in CBF. An important observation from a parallel study (*study 2*) was that cerebral perfusion gauged by MCA *V*_mean_ declined when control (euhydration) exercise was continued to exhaustion and core temperature remained at normothermic levels (Fig. 5). Taken together, the findings from both studies indicate that CBF declines during exhaustive exercise regardless of the hydration status and the level of core temperature, yet the maintenance of euhydration and stable core temperature slows the rate of the CBF decline late in fatiguing exercise. The CBF decline with dehydration is therefore closely coupled to factors associated with the fatigue processes rather than reduced body fluids or core hyperthermia per se.

Another pertinent finding was that blood flow to extracranial tissues displayed a distinct temporal dynamic response to that of the cerebral circulation but was blunted late in exercise in the dehydrated condition. These findings agree with reports of two- to threefold increases in ECA blood flow in response to passive heat stress (2, 36) and incremental exercise (40). The ECA supplies blood to the skin circulation of the face and neck. In this light, the enhanced ECA perfusion may be part of circulatory adjustments required to meet the thermoregulatory demands for heat transfer to the environment surrounding the head (36). Collectively, these data suggest that dehydration accentuates the rise in internal temperature, reduces extracranial blood flow at point of exhaustion, accelerates the decline in cerebral perfusion, and leads to early exhaustion during prolonged exercise in the heat.

#### Regional head blood flow regulation.

Both local and systemic factors have been implicated in the regulation of CBF through modulation of vascular conductance (or resistance) and cerebral perfusion pressure. The decline CBF in the dehydration trial was accompanied by a falling cerebrovascular conductance, indicative of augmented vasoconstriction. Changes in blood gas variables (48) and sympathetic activity (28) may play a role in the control of CBF during conditions including exercise. In particular, CO_2_ is a potent vasoactive substance within the cerebral vasculature, with reductions in Pa_CO_2__ inducing cerebral vasoconstriction and increases leading to vasodilation (2, 25, 48). The decline in Pa_CO_2__ is attributed to hyperventilation with increasing exercise intensity and a rising core body temperature (46, 47). Thus, the decline in vascular conductance with dehydration was associated with reductions in Pa_CO_2__ (*R*^2^ = 0.44, *P* < 0.01; Fig. 4*C*) and cerebral perfusion pressure but unrelated to the stable arterial and internal jugular venous O_2_ variables (data not shown). Pa_CO_2__, however, accounted for only half of the variance in vascular conductance, and there was only a modest relationship between CBF and cerebral perfusion pressure (*R*^*2*^ = 0.18, *P* < 0.05). Another potential contributing factor is enhanced sympathetic activity. The cerebral vasculature is richly innervated with sympathetic nerves, and observations of NE spillover into the internal jugular venous outflow, as seen here with dehydration, may reflect sympathetic-mediated vasoconstriction of the cerebral vessels (28, 50). However, despite increases in arterial and jugular venous NE concentration, the relationships between cerebrovascular tone and plasma NE were weak (43, 46a, 49).

The distinct dynamics of the extracranial circulation might involve different regulatory mechanisms. There was a close coupling between the increase in ECA blood flow/conductance and the rise in internal jugular blood temperature (*R*^*2*^ = 0.64, *P* < 0.01; Fig. 4*D*). We did not investigate the control of skin blood flow, but cutaneous blood flow is enhanced by elevated local and core temperatures (5, 21, 39). The role of sympathetic activity in the response to local and internal temperature changes is substantiated by an elevation in skin sympathetic nerve activity (7, 32), promoting cutaneous blood distribution and sudomotor function (5, 24). With a similar ECA profile in both trials, it is more likely that exercise per se attenuates cutaneous perfusion as rising internal temperature (above 38°C) is not matched by further increases in skin perfusion, as measured in the forearm (4, 17). Dehydration also limits maximal skin perfusion during exercise (30) and enhances systemic vascular resistance (16), leading to attenuated cutaneous blood flow (23). Taken together, these findings support that blood flow to the extracranial vascular bed is influenced by local and internal temperatures and enhanced sympathetic activity.

#### Impact of dehydration on cerebral metabolism.

**Fig. 6.**
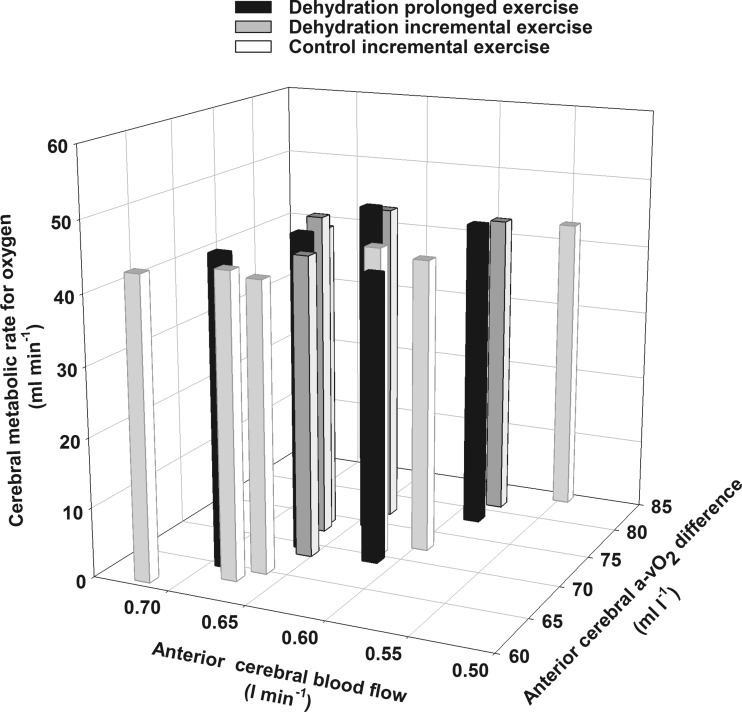
Anterior arterial to internal jugular venous O_2_ difference, anterior cerebral blood flow, and CMR_O_2__ during prolonged and incremental exercise. Values are means ± SE during prolonged (*n* = 9) and incremental (*n* = 8) exercise. Baseline and incremental exercise to exhaustion data in control and dehydrated conditions have been previously reported (44). An inverse relationship between changes in blood flow and a-v O_2_ differences is shown (all points; *R*^2^ = −0.29, *P* = 0.01). Data are from a variety of exercise intensities, exercise durations, hydration status, and rest-to-exercise transitions where CMR_O_2__ remained stable at ∼ 45 ml/min.

A reduction in O_2_ supply can compromise organ metabolism, as shown in contracting skeletal muscle with significant hyperthermia during maximal exercise (13). Here, we asked whether prolonged fatiguing exercise and dehydration compromise cerebral metabolism. Similar to findings during maximal exercise (44), the decline in CBF was met by an equal increase in O_2_ extraction such that CMR_O_2__ was maintained during prolonged exercise in the dehydrated state, in agreement with other independent metabolic measures obtained in this study. In addition, glucose uptake, cerebral lactate exchange, OGI, and OCI remained unchanged, and the cerebral respiratory quotient (∼1.03) was stable (8). There is contrasting evidence that CMR_O_2__ might be elevated during strenuous exercise combined with severe hyperthermia compared with control exercise (33), thereby arguing that the metabolic demand of the brain increases during exhaustive exercise. This conclusion, however, is based on two data points. To provide a more comprehensive account of the cerebral metabolic responses to exercise and establish whether CMR_O_2__ is altered during fatiguing exercise, we plotted the anterior CBF and arterial to venous O_2_ difference data from the current prolonged exercise protocol together with the reported baseline and incremental exercise data obtained in the same individuals (Fig. 6) (44). This analysis showed that CMR_O_2__ remained stable across a variety of exercise intensities, exercise durations, hydration conditions, and rest-to-exercise transitions, as variations in CBF were met by proportional changes in O_2_ extraction. The unchanged global cerebral metabolism may reflect a balance of regional alterations in metabolic (and thus flow) demand. In this construct, exercise of increasing intensity both augments and downregulates regional blood flow across the brain (9). It remains to be clarified whether regional cerebral hypoperfusion, particularly that assessed in the frontal cortex, reflects an important process leading to fatigue or a selective downregulation in neuronal activity in regions of the brain that are less important during physical exertion (10). Restoration of CBF and cerebral oxygenation during strenuous exercise in the heat does not affect the development of fatigue (22). Therefore, although regional differences might exist, our data suggest that metabolic activity of the brain as a whole is neither enhanced nor compromised during exhaustive exercise in trained healthy individuals.

#### Experimental considerations.

Ultrasound imaging of extracranial vessels and MCA *V*_mean_ are appropriate for assessing CBF during dynamic exercise in an upright position (18, 40, 44). Notwithstanding, some flow is directed to branch vessels (e.g., the choroidal artery), but this is unlikely to impact on the measured CBF during exercise (27). Furthermore, changes in MCA *V*_mean_ may underestimate cerebral perfusion as vessel diameter changes are unknown. However, during exercise, changes in MCA *V*_mean_ are similar to absolute blood flow measured in the ICA (18). Blood samples were obtained from the left internal jugular vein and asymmetry may be present in the venous drainage of the brain (42). However, blood gas variables obtained from the left and right internal jugular vein are similar both at rest and during exercise (11, 20). We were unable to obtain internal jugular venous blood samples in the control trial, but it has been demonstrated that leg and systemic arterial to venous O_2_ differences are stable when euhydration is maintained during prolonged exercise in the heat (12). We therefore expect that arterial to venous O_2_ differences across the brain would also be unchanged when CBF is not compromised. Finally, the present studies used only male participants. Previous studies using both male and female participants have not demonstrated any sex differences in CBF responses to exercise (40).

#### Conclusions.

Dehydration leads to augmented cerebrovascular strain, as indicated by blunted cerebral and extracranial perfusion during prolonged exercise in the heat. However, despite the circulatory strain associated with fatigue, secondary to dehydration and hyperthermia, cerebral metabolism is not impaired due to compensatory increases in O_2_ and substrate extraction across the brain. The findings imply that reduced cerebral metabolism is unlikely to explain the reduced exercise capacity with dehydration during prolonged exercise in the heat. In contrast, both a reduction in CBF and extracranial perfusion may influence fatigue.

## GRANTS

This work was supported by a grant from the Gatorade Sports Science Institute, PepsiCo Incorporated.

## DISCLAIMERS

The views contained within this document are those of the authors and do not necessarily reflect those of PepsiCo Incorporated.

## DISCLOSURES

No conflicts of interest, financial or otherwise, are declared by the author(s).

## AUTHOR CONTRIBUTIONS

Author contributions: S.J.T., N.H.S., and J.G.-A. conception and design of research; S.J.T., S.T.C., I.L., B.G., K.K., N.H.S., and J.G.-A. performed experiments; S.J.T., S.T.C., I.L., B.G., K.K., and J.G.-A. analyzed data; S.J.T., S.T.C., I.L., B.G., K.K., N.H.S., and J.G.-A. interpreted results of experiments; S.J.T. prepared figures; S.J.T. drafted manuscript; S.J.T., S.T.C., I.L., B.G., K.K., N.H.S., and J.G.-A. edited and revised manuscript; S.J.T., S.T.C., I.L., B.G., K.K., N.H.S., and J.G.-A. approved final version of manuscript.
